# The Functional and Morphological Changes of the Cervical Intervertebral Disc after Applying Lordotic Curve Controlled Traction: A Double-Blind Randomized Controlled Study

**DOI:** 10.3390/ijerph16122162

**Published:** 2019-06-19

**Authors:** Chang-Hyung Lee, Sung Jin Heo, So Hyun Park, Hee Seok Jeong, Soo-Yeon Kim

**Affiliations:** 1 Rehabilitation Medicine, Pusan National University School of Medicine and Research Institute for Convergence of Biomedical Science and Technology, Pusan National University Yangsan Hospital, Yangsan 50612, Korea; aarondoctor@gmail.com; 2 Research Institute for Convergence of Biomedical Science and Technology, Pusan National University Yangsan Hospital, Yangsan 50612, Korea; whitegusdl@hanmail.net; 3 Department of Physical Therapy, Youngsan University, Yangsan 50510, Korea; ptpsh@ysu.ac.kr; 4 Radiology Medicine, Pusan National University School of Medicine and Research Institute for Convergence of Biomedical Science and Technology, Pusan National University Yangsan Hospital, Yangsan 50612, Korea; mediknight@hanmail.net

**Keywords:** traction, cervical lordosis, intervertebral disc, pain, function, morphology

## Abstract

The newly developed cervical lordotic curve-controlled traction (C-LCCT) appears to be an ideal method to improve the treatment outcome in patients with cervical intervertebral disc disease. The purpose of this study was to investigate the treatment outcomes of C-LCCT including the functional and morphological changes of the cervical intervertebral disc compared to traditional traction (TT) with a randomized controlled trial design. A total of 40 patients with cervical intervertebral disc disease at the C5/6 level confirmed by magnetic resonance imaging were recruited and assigned to either the C-LCCT group or the TT group. The comprehensive health status changes of the patients were recorded using pain and functional scores (Visual Analogue Scale, Oswestry Disability Index) and morphological changes (cervical lordosis, cervical central canal area) before and after the traction treatment. Both groups showed a significant improvement in pain scores after traction (*p* < 0.05). The functional score and morphological changes improved significantly after treatment in the C-LCCT group. However, there was no significant improvement in the TT group (*p* < 0.05). The C-LCCT showed significant pain, functional, and morphological improvement compared to TT. C-LCCT could be effective in improving the treatment outcomes of the traction technique in patients with cervical intervertebral disc disease.

## 1. Introduction

Cervical traction has often been used as a conservative method to treat patients with neck pain in clinical settings [1]. A variety of theories in previous reports have suggested the positive effects of the traction technique [2,3,4,5]. For example, it increases circulation in the cervical blood vessels by stretching the paraspinal muscles and ligaments and facilitates muscle relaxation. Additionally, it decreases nerve root compression by distracting the vertebrae and expanding the intervertebral foramen [6,7]. Traction also appears to reduce pain transmission in the sensory fibers of the spinal cord by stimulating the large afferent fibers of joints and muscles in the presynaptic space [8]. Therefore, it can lead to pain relief, increased cervical range of motion, and improved functional status by elongating the intervertebral spaces [9,10,11]. Nonetheless, a number of studies have reported mixed results of the traction technique and shown that traction has no advantage over other conservative methods, such as physical treatment and placebo intervention, for cervical intervertebral disc diseases [12,13,14,15]. Some studies showed pain reduction and functional improvement following traction. However, other studies reported no changes after applying traction. Most studies reported the treatment outcomes by comparing pain and health status changes with other conservative methods. In addition, no study measured any significant morphological improvements—such as decreased severity of herniated disc or magnetic resonance imaging (MRI) changes—after traction. Several systematic reviews reported that the efficacy of cervical traction was inconclusive due to poor methodology and technical failures [12,16,17]. Due to the lack of controlled treatment guidelines, patient selection, measurement method, and controlled study design, the precise effect of traction could not be observed in previous studies. Therefore, the clinical application of traction for the management of cervical intervertebral disc disease is not popular, which underscores the need for further theoretical and technical analyses.

Improper pressure loading on disc structures during the traditional traction (TT) technique may result in poor clinical outcomes. In the supine position, the lordotic curve is decreased under the effect of gravity on the cervical curve. When traction force is applied to the cervical spine in the supine position, it primarily straightens the natural lordotic curve rather than decompressing the intervertebral discs. Under TT, the posterior spinal structures, such as facet joints, and posterior longitudinal and interspinous ligaments are elongated more so than the anterior spinal structures [16]. When traction pressure is applied to the spine in the supine position using TT, the lordotic curve decreases following substantial elongation of the posterior spinal structures, resulting in pain. Thus, we investigated whether the traction force against the vertebrae maintains the lordotic curve while distributing the force equally to the anterior and posterior elements of the spinal structure. Subsequently, a newly designed cervical lordotic curve-controlled traction device (C-LCCT) was invented. In our previous study, we measured the therapeutic effect of LCCT on patients with lumbar intervertebral disc disease (under review). Subsequently, we applied this technique to patients with cervical intervertebral disc disease. We tried to analyze the morphological changes of cervical disc severity (cervical lordosis and cervical central canal area) using plain radiography (X-ray) and MRI following application of the newly developed C-LCCT. Thus, the purpose of our study was to compare the clinical and morphological changes after application of C-LCCT and TT in patients diagnosed with cervical intervertebral disc disease. We conducted a double-blind randomized controlled study to analyze the patients’ pain, functional, and morphological disc changes after traction.

## 2. Materials and Methods 

In this prospective double-blind randomized controlled study, 40 patients were conveniently selected from the outpatient clinic. All patients gave informed consent for inclusion before they participated in the study. The study was approved by the institutional review board (IRB 04-2018-034).

### 2.1. Study Population and Sample

A total of 40 patients with cervical intervertebral disc disease on the C5/6 level confirmed by MRI were recruited from April 2018 to January 2019. The severity of the cervical disc disease was measured prior to inclusion based on the Visual Analogue Scale (VAS), Oswestry Disability Index (ODI), and Cobb angle on a lateral view of cervical spine using X-ray and central canal area using cervical MRI. The inclusion criterion was a diagnosis of C5/6 nerve root compression by cervical MRI and/or cervical radiculopathy confirmed by Electomyography (EMG), with lasting cervical pain of more than three months. Exclusion criteria were as follows: (1) central disc protrusion with cervical spinal cord compression; (2) acute inflammation; (3) malignant disease; and (4) unstable vertebrae or previous history of operation on spine. Patients were randomly assigned to two groups of C-LCCT and TT by a research physician using a computer-generated table of random numbers. None of the patients was aware of their group. The evaluations were carried out by a physician who was blinded to the treatment.

### 2.2. C-LCCT Versus TT

**Figure 1:**
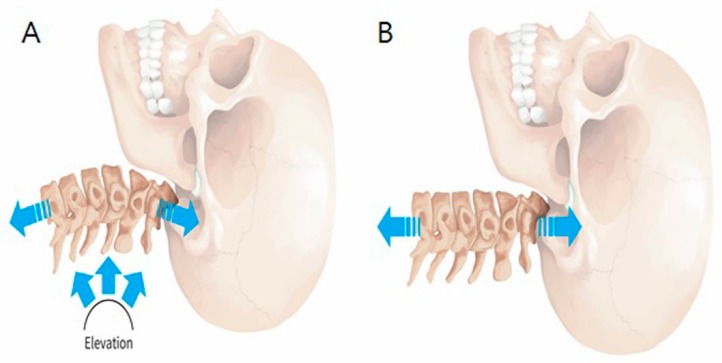
Technique of cervical traction: (**A**) cervical lordotic curve-controlled traction (C-LCCT) and (**B**) traditional traction (TT).

The C-LCCT (KINETRAC KNX-9900, Hanmed Co., Gimhae, Republic of Korea) was designed to maintain the natural lordotic curve by supporting the cervical curve at the C4–6 intervertebral disc space. The C-LCCT and TT methods are compared in Figure 1. Initially, a magnetic marker was attached to the C4–6 intervertebral disc space by physical palpation. An automated tracking system also ensured the cervical lordotic curve by elevating C4–6, using this marker during C-LCCT. During C-LCCT, the height of the elevated cervical lordotic curve support was increased to the most comfortable point for each participant. Conversely, the TT method was administered as usual without maintaining the cervical lordotic curve during traction. In this case, patients remained in a supine position on the traction table with knee supports placed under each knee. Traction was applied and gradually increased to the maximal tolerable level or until the force reached a third of the patient’s weight for 15 minutes in one session. Three sessions per week for five weeks for a total of 15 sessions were conducted.

### 2.3. Outcome Measurement Using Pain and Functional Status 

Changes to functional improvements and pain scores of participants were measured before the first intervention and after the last treatment session. 

The ODI consists of 10 items that refer to activities of daily living that might be disrupted by pain. The items are as follows: 1. Pain intensity, 2. Personal care, 3. Lifting, 4. Walking, 5. Sitting, 6. Standing, 7. Sleeping, 8. Sex life, 9. Social life, and 10. Traveling. Each item is rated on a 6-point Likert scale. The total score is transferred onto a scale ranging from 0 to 100, where 0 indicates no disability and 100 indicates the worst possible disability [18]. 

Pain in the trunk and lower extremities exacerbated during activities of daily living was measured using the VAS, which ranges from 0–10. The patients were asked to place a mark along the line to denote their pain level; 0 reflected “no pain” and 10 reflected the “worst pain” [19].

### 2.4. Morphological Changes in Cervical Disc Severity 

#### 2.4.1. Cervical Lordotic Curve

**Figure 2:**
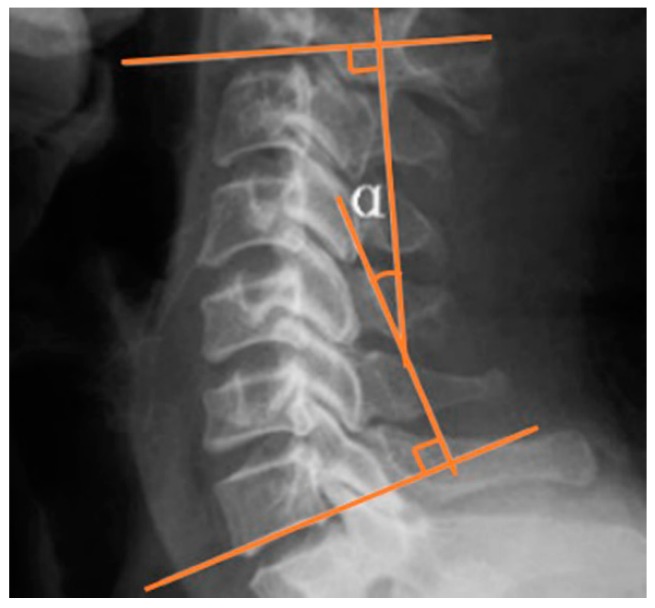
Cobb angle of cervical lordotic curve.

As shown in Figure 2, the Cobb angle was calculated by measuring the angle between the superior border of the C2 vertebral body and the inferior border of the C7 in neutral position in lateral view of cervical spine X-ray.

#### 2.4.2. Central Canal Area Assessment 

**Figure 3:**
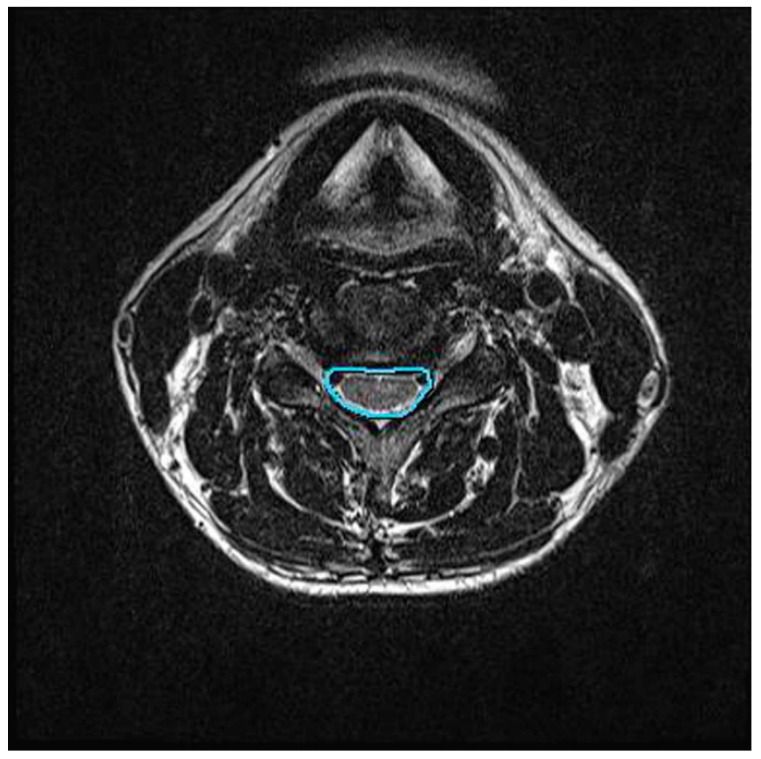
Central canal area in axial view of cervical spine MRI.

We assessed the changes in the area of the cervical central canal before and after cervical traction. Using the MR T2-weighted images, the axial image with the greatest disc level associated with the greatest neurologic compression was selected for measurement. In this study, the C5/6 intervertebral disc level was selected. Digital measurement of the central canal area outline was performed by tracing the boundaries of the dural cross-sectional area in the axial MRI at the C5/6 disc level (Figure 3). Measurements were conducted by a single-blinded radiologist with more than 10 years of experience. Each measurement was repeated three times by the same radiologist to enhance repeatability.

The evaluations of pain (VAS), function (ODI), and morphology (cervical spine plain radiography, cervical spine MRI) were conducted within two days before the first treatment session and after the last treatment session.

### 2.5. Statistical Analysis: Sample Size Determination 

Prior power calculations indicated that 18 patients were needed in each group to detect differences in cervical neck pain between the groups at 80% power and 5% significance. A two-tailed test was performed with an expected effect size of d = 0.8 based on a pilot study of 10 patients who underwent the same intervention between November 1, 2017, and January 28, 2018. Data were analyzed using a paired t-test and independent t-test. The significance level was set at *p* < 0.05. SPSS software ver. 22.0 (IBM Corp., Armonk, NY, USA) was used for all statistical analyses. 

## 3. Results

The demographic data of the patients are presented in Table 1. All participants completed the study without dropouts. The C-LCCT and TT groups included 13 females and seven males (mean age 48.8 ± 13.3) and 15 females and five males (mean age 43.2 ± 16.2), respectively. The two-sample t-tests revealed no statistically significant differences between the two groups in gender, age, height, weight, BMI, duration of cervical pain, VAS, or ODI. No significant adverse events or symptom aggravation was observed after treatment in either group.

### 3.1. Pain and Functional Status Measurement 

The pain and functional changes are presented in Table 2. After 15 sessions of treatment, the analysis of covariance revealed a significant difference between the C-LCCT and TT groups. In pain and functional measurement, the C-LCCT group showed superior outcomes compared to the TT group. Both groups showed a significant decrease in VAS pain score. The changes in ODI were significantly higher in the C-LCCT group. However, there was no significant change in ODI in the TT group.

### 3.2. Morphological Change Measurement: Cobb Angle and Central Canal Aarea 

The morphological changes after treatment are shown in Table 3. The Cobb angle of the C-LCCT group at C2–7 was significantly increased after treatment (4.8° ± 10.9 and 16.9° ± 12.7, before and after treatment, respectively). Conversely, the TT group showed no significant difference in the Cobb angle after treatment at C2–7(5.2° ± 8.6, 4.9° ± 9.8). Rather, cervical lordosis was decreased slightly.

The change of the central canal area in cervical spine MRI was significantly greater in the C-LCCT group (*p* < 0.01). However, there was no significant change in the central canal area in the TT group after treatment (*p* > 0.05).

Both groups showed significant decreases in pain score. However, changes in functional score, Cobb angle, and central canal area improvement were significantly greater in the C-LCCT group than the TT group (Table 4).

## 4. Discussion

Traction has been used as one of the ideal conservative methods to elongate the intervertebral disc space [20,21]. In spite of the theoretical benefits associated with traction, its clinical outcomes have not been superior to those of other conservative treatments. Systematic reviews have provided little support for traction in neck pain management [17,22].

In addition to the lack of well-designed double-blind controlled studies, different techniques, guidelines, and indications have also resulted in the poor use of traction clinically. The various traction methods performed by physical therapists at different levels of expertise, including the indications and techniques, delivery modes and parameters, and concomitant use of supplemental interventions, have all greatly influenced the treatment outcomes. One study reported that 93.1% of the therapists were willing to use traction in patients with cervical nerve root compression [4]. However, traction did not yield consistent and successful outcomes, and it often required supplementation with other conservative methods [12].

Unfortunately, most studies concluded that the use of cervical traction was consistent only with proposed criteria that identify patients who are likely to benefit from the intervention [23]. Despite its poor reputation, various techniques for conducting traction and guidelines should be regulated for optimal therapeutic outcomes when used in conjunction with multiple interventions. 

Based on previous studies, traction may be regarded as one possible option to treat cervical nerve root compression. However, it is not a strong and reliable method to relieve compression. The authors addressed the technical limitations of the traditional type of traction technique to ensure successful outcomes. Due to the force of gravity during the supine position, improper pressure distribution decreases the lordotic curve of the spine. In addition to the effect of positional gravity in the supine posture, the linear traction also decreases the lordotic curve. As a result, despite the fact that traction increases and elongates the intervertebral disc space, the TT approach decreases the lordotic curve, resulting in excessive pressure on the posterior spinal structures. If the TT force exceeds the resistance limit of the patients, it stretches the posterior spinal structures, resulting in pain and poor outcomes. 

The C-LCCT approach discussed in this study maintains the lordotic curve during traction via morphological improvement, resulting in effective outcomes with balanced traction force distribution on the spinal structure (Figure 1). In addition, an automated tracking system also ensures the cervical lordotic curve by elevating C4–6, using this marker during C-LCCT. This automatic tracking system maintains the precise lordotic curve during C-LCCT. Therefore, equal elongation of the intervertebral space can be ensured during the whole traction session. 

In our study, the therapeutic effect of C-LCCT was significantly greater than that of TT in terms of pain, functional, and morphological outcomes. Both groups showed significant improvement in pain after treatment (Table 2). However, there was no significant change in functional and morphological scores of patients treated with TT. A significant change in the central canal area was observed in patients treated with C-LCCT (130.9 ± 40.5 and 136.0 ± 43.2, *p* < 0.001 *, before and after treatment, respectively). The equal distribution of traction force in C-LCCT (anteriorly and posteriorly) ideally elongates the intervertebral disc space without generating needless muscle tension and thereby increases its efficiency. Based on our results, maintenance of the lordotic curve is essential to improve the therapeutic outcomes in patients treated with TT.

Despite the favorable results, our study has several limitations. First, various conditions associated with spinal nerve compression were not included in this study. The severity of nerve root compression and the structural diversities may influence treatment outcomes. Further studies investigating various conditions of disc diseases are needed. Second, a long-term clinical study is desirable to corroborate the preliminary findings of short-term efficacy at five weeks after treatment. Studies establishing the long-term effects of C-LCCT will clarify the value of the clinical application of traction.

Consistent with our original hypothesis that appropriate traction force distribution is key to maintaining the lordotic curve, we found significant successful outcomes using C-LCCT. Compared to TT, our randomized double-blind controlled study showed statistically significant differences in pain, functional scores, and morphological changes. Additional samples including diverse patient groups and modified treatment guidelines are needed to generalize our results. Based on our results, the newly invented C-LCCT is recommended as a reliable and conservative method to alleviate spinal nerve compression in patients diagnosed with cervical intervertebral disc disease.

## 5. Conclusions

Maintenance of the lordotic curve appears to be one of the key factors to improve treatment outcomes of cervical traction in patients with cervical nerve root compression. Based on our results, we suggest C-LCCT as a reliable treatment option available under clinical settings.

## Figures and Tables

**Table 1:** Demographic characteristics of participants.

Variables	All (*n* = 40)	C-LCCT (*n* = 20)	TT (*n* = 20)	*p* Value
Age (years)	46 ± 14.6	48.8 ± 13.3	43.2 ± 16.2	0.684
Height (cm)	165.0 ± 8.2	163.6 ± 7.5	167.6 ± 9.2	0.628
Weight (kg)	63.3 ± 11.4	62.4 ± 11.2	64.9 ± 12.0	0.713
BMI	23.1 ± 3.2	23.2 ± 3.4	23.0 ± 3.0	0.669
Duration of cervical pain (mean, months)	15.5 ± 13.4	16.5 ± 12.8	13.5 ± 15.1	0.541
Initial VAS	6.7 ± 0.4	6.8 ± 0.8	7.0 ± 0.0	0.852
Initial ODI (%)	29.2±10.2	29.9 ± 15.7	28.5 ± 4.6	0.788

All values represent mean ± standard deviation; BMI: Body mass index; VAS, Visual analogue scale (0 = no pain; 10 = worst pain ever); ODI, Oswestry Disability Index (0 = no disability; 100 = maximum disability possible).

**Table 2:** Pain and functional scores in C-LCCT versus TT group.

Variables	Before Treatment	After Treatment	*p* Value
A. C-LCCT group			
VAS	6.78 ± 0.8	3.33 ± 0.8	<0.001 *
ODI (%)	29.85 ± 15.6	20.15 ± 11.8	0.003 *
B. TT group			
VAS	7.0 ± 0.0	4.27 ± 0.9	0.006 *
ODI (%)	28.48 ± 4.6	26.87 ± 11.1	0.470

All values represent mean ± standard deviation; VAS, Visual Analogue Scale (0 = no pain; 10 = worst pain ever); ODI, Oswestry Disability Index (0 = no disability; 100 = maximum disability possible); * *p* < 0.05.

**Table 3:** Morphological changes (Cobb angle and central canal area) in C-LCCT versus TT group.

Variables	Before Treatment	After Treatment	*p* Value
A. C-LCCT group			
Cobb angle at C2–7 (°)	4.8 ± 10.9	16.9 ± 12.7	<0.001 *
Central canal area (mm^2^)	130.9 ± 40.5	136.0 ± 43.2	<0.001 *
B. TT group			
Cobb angle at C2–7 (°)	5.2 ± 8.6	4.9 ± 9.8	0.781
Central canal area (mm^2^)	137.9 ± 37.6	136.7 ± 41.4	0.549

* *p* < 0.05.

**Table 4:** Comparison of changes between C-LCCT and TT groups.

Variables	C-LCCT	TT	T	*p* Value
VAS	−2.7 ± 1.5	−2.7 ± 1.0	−1.6	>0.05
ODI (%)	−9.7 ± 9.8	−1.6 ± 7.1	−2.3	<0.05 *
Cobb angle	10.1 ± 4.5	−0.3 ± 5.3	7.6	<0.001 *
Central canal area (mm2)	5.1 ± 5.3	−2.5 ± 5.8	3.7	<0.001 *

* *p* < 0.05.
